# Part-Based Visual Tracking via Online Weighted P-N Learning

**DOI:** 10.1155/2014/402185

**Published:** 2014-07-15

**Authors:** Heng Fan, Jinhai Xiang, Jun Xu, Honghong Liao

**Affiliations:** ^1^College of Engineering, Huazhong Agricultural University, Wuhan 430070, China; ^2^College of Science, Huazhong Agricultural University, Wuhan 430070, China; ^3^Department of Physics, Central China Normal University, Wuhan 430079, China; ^4^School of Computer Science and Technology, Huazhong University of Science and Technology, Wuhan 430074, China

## Abstract

We propose a novel part-based tracking algorithm using online weighted P-N learning. An online weighted P-N learning method is implemented via considering the weight of samples during classification, which improves the performance of classifier. We apply weighted P-N learning to track a part-based target model instead of whole target. In doing so, object is segmented into fragments and parts of them are selected as local feature blocks (LFBs). Then, the weighted P-N learning is employed to train classifier for each local feature block (LFB). Each LFB is tracked through the corresponding classifier, respectively. According to the tracking results of LFBs, object can be then located. During tracking process, to solve the issues of occlusion or pose change, we use a substitute strategy to dynamically update the set of LFB, which makes our tracker robust. Experimental results demonstrate that the proposed method outperforms the state-of-the-art trackers.

## 1. Introduction

Object tracking is one of the most important components of many applications in computer vision, such as human computer interactions, surveillance, and robotics. However, robust visual tracking is still a challenging problem, which is affected by partial or full occlusion, illumination, scale, and poses variation [1]. The key for the object tracking is to construct an effective appearance model. Many tracking algorithms have been proposed recently, but designing a robust appearance model is still a major challenge, which is affected by both extrinsic (e.g., illumination variation, background clutter, and partial or full occlusion) and intrinsic (e.g., scale and pose variation) factors. In order to handle these problems, a wide range of appearance models based on different visual representations and statistical modeling techniques have been presented by researchers. In general, these appearance models can be categorized into two types: appearance model based on visual representation, such as global-based representation [2–6] and local-based representation [7–11]; appearance model based on statistical modeling, such as generative model [12–16] and discriminative model [5, 6, 17–20].

In this paper, we propose a part-based visual tracking algorithm with online weighted P-N learning. Weighted P-N learning is first proposed by assigning weights (property weight and classification weight) to each sample in training sample set, which can decrease false classification and improve the discriminative power of classifier. Then, we segment object into fragments and select parts of them as local feature blocks to represent the object. Finally, we train classifier for each LFB with weighted P-N learning to obtain the corresponding classifier, respectively, and track each LFB independently within the framework of Lucas-Kanade optical flow [21]. During tracking process, a real-time valid detection method is used for each LFB. If certain LFB is invalid, we use a replacing strategy to update the local feature block set, which can ensure successful tracking.

*Contributions*. The contributions of this paper include the following.

- A part-based visual tracking algorithm with online weighted P-N learning is proposed in this work. Object is represented by LFBs and tracked. When occlusion or distortion happens, a strategy is adopted to replace invalid LFB and keep the new LFB set effective.
- We define the weights (property weight and classification weight) for each sample in training process of P-N learning.
- An online weighted P-N learning is presented by assigning weight to each sample in training sample set, which can improve discriminative power of classifier by decreasing classification errors and increasing the accuracy of tracker.

The rest of the paper is organized as follows.  Section 2 reviews the related work of this paper.  Section 3 introduces weighted P-N learning. Proposed tracking method is presented in detail in Section 4. Experimental results are shown in Section 5.  Section 6 concludes the whole paper.

## 2. Related Work

Recently, many trackers based on local feature representation have been proposed. Adam et al. [8] present a fragment-based tracking approach, and further, Wang et al. [22] embed the fragment-based method into mean shift tracking framework. This tracking method estimates the target based on voting map of each part via comparing its histogram with the template's. Nevertheless, static template with equal importance being assigned to each fragment obviously lowers the performance of tracker. In order to overcome the shortcomings, Jia et al. [7] propose a fragment-based tracking method using online multiple kernel learning (MKL). All the patches are assigned to different weights based on the importance learned by MKL. However, this strategy may still cause drifting problem. Occlusion especially, which makes part patches invalid, leads to errors in computing voting map, even tracking failure. Wang et al. [10] introduce a tracking method based on superpixel. It only computes the probabilities of superpixels belonging to target, which is prone to drift away in color-similar background and whose tracking results will shrink to the unoccluded part of object when occlusion happens.

Another type of tracking method is based on discriminative appearance model. Tang et al. [23] present a tracking method based on semisupervised support vector machines. This tracker employs a small number of labeled samples for semi-supervised learning and develops a classifier to mark the unlabeled data. Babenko et al. [5] propose a multiple instance learning (MIL) method for visual tracking. This approach solves the problem of slight inaccuracies in the tracker leading to incorrectly labeled training samples and can alleviate drift problem to some extent. However, the MIL tracker might detect positives, which are less important because they do not consider the importance of sample in learning process. Further, Zhang and Song [20] suggest a weighted multiple instance learning (WMIL) tracking method. It assigns weight to each sample based on the corresponding importance. This approach improves the robustness of tracker. Kalal et al. [17] propose a method called P-N learning, which learns from positive samples and negative samples, to construct a classifier. In the meanwhile, the discriminative properties of classifier are improved by two categories of constrains that are termed P-constrains and N-constrains. However, false classification in P-N learning degrades the classifier in some degree.

Another work similar to ours is [9], which utilizes blocks to represent the object. However, the blocks are easily invalided when target appearance changes, which undermines its robustness to nonrigid distortion or occlusion. In our work, we employ LFBs to represent target and use a dynamically updating mechanism to update the local feature block set, which guarantees each LFB in the set is valid when occlusion or deformation occurs. Hence, our tracker is more robust and effective.

## 3. Weighted P-N Learning

**Figure 1:**
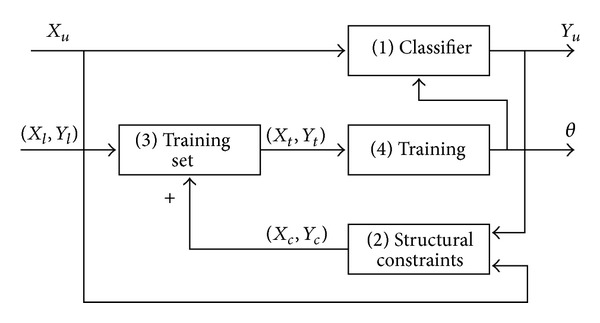
A flowchart in [17] to explain P-N learning. P-N learning algorithm initially develops a classifier from prior knowledge and then iterates over: (1) classify the unlabeled data and label it; (2) reclassify the samples within constrains and label them; (3) expand the training sample set; (4) retrain the classifier.

P-N learning is a semisupervised online learning algorithm proposed by Kalal et al. [17, 24–26]. Let *x* be a sample in feature space *X*, and let *y* be a label in label space *Y* = {1, −1}. A set of samples *X* and corresponding set of labels *Y* are defined as (*X*, *Y*), which is termed a labeled set. The aim of P-N learning is to develop a binary classifier *f* : *X* → *Y* based on a priori labeled set (*X*_*l*_, *Y*_*l*_) and improve its discriminative performance by unlabeled data *X*_*u*_. The flowchart of P-N learning approach in [17] is shown in Figure 1.

### 3.1. Classifier Bootstrapping

The binary classifier *f* is a function parameterized by *θ*. Similar to supervised learning, P-N learning is to estimate parameter *θ* via training sample set (*X*_*t*_, *Y*_*t*_). Nevertheless, it is worth noticing that the training set is iteratively expanded through adding samples, which is screened by constraints from unlabeled data. Initially, classifier and its parameter *θ* ^0^ are obtained by training labeled samples. Then, the process proceeds iteratively. In iteration *k*, all the unlabeled samples are marked by classifier in iteration  *k* − 1; namely,

(1) $$\begin{array}{c} y_{u}^{k}=f\left(x_{u}\;|\;\theta^{k-1}\right),\;x^{u}\in X_{u}. \end{array}$$

Then, the constraints are utilized to revise the classification results and add the corrected labels to training set. Iteration *k* ends with retraining classifier by using the renewed training sample set.

During training process, the classifier may identify the unlabeled data with wrong labels. Any sample may be screened many times with constraints and hence can be represented mistakenly in the training set repeatedly. Obviously, it can significantly degrade discriminative performance of classifier and therefore lower the accuracy of tracking. In order to further improve the accuracy and robustness of the classifier, we propose a weighted P-N learning method by assigning weight to each sample in training set. Sample *j* in training set has two categories of weight which are termed P-weight *W*_*j*_^+^ and N-negative *W*_*j*_^−^. P-weight represents the probability of being a positive sample, and N-weight represents the probability of being a negative sample. In iteration *k*, sample *j* from training set is represented as positive sample for *C*_*j*_^+^ times and as negative sample for *C*_*j*_^−^ times. The positive weight *W*1_*j*_^+^ and negative weight *W*1_*j*_^−^ are determined by the following formulation:

(2) $$\begin{array}{c} W1_{j}^{+}=\frac{C_{j}^{+}}{C_{j}^{+}+C_{j}^{-}},\\ W1_{j}^{-}=\frac{C_{j}^{-}}{C_{j}^{+}+C_{j}^{-}}. \end{array}$$

Besides, the probability of sample *j* being positive or negative in training set obtained by classifier is defined as classification weights *W*2_*j*_^+^ and *W*2_*j*_^−^ (in Section 3.3). The P-weight and N-weight of sample *j* can be then obtained by the following formulation:

(3) $$\begin{array}{c} W_{j}^{+}=W1_{j}^{+}+W2_{j}^{+},\\ W_{j}^{-}=W1_{j}^{-}+W2_{j}^{-}. \end{array}$$

At last, sample *j* is determined to be either positive or negative via the following formulation:

(4) $$\begin{array}{c} \frac{W_{j}^{+}}{W_{j}^{-}}\geq 1\;\text{positive sample,}\\ \frac{W_{j}^{+}}{W_{j}^{-}}<1\;\text{negative sample.} \end{array}$$

**Figure 2:**
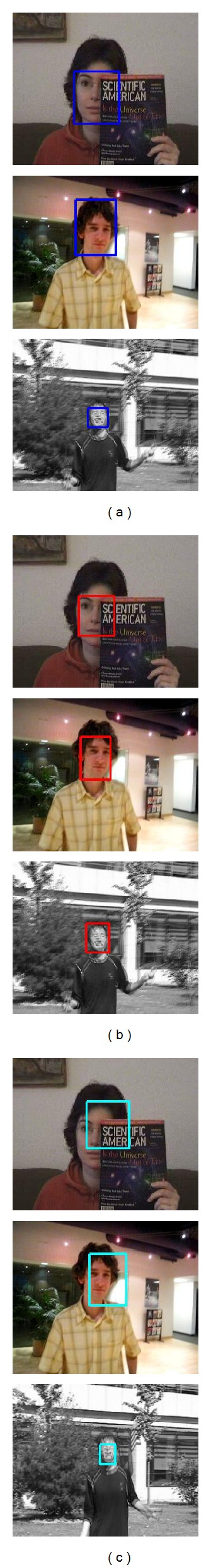
(a) Ground truth. (b) Tracking results with weighted P-N learning. (c) Tracking results based on P-N learning.

Figure 2 demonstrates the tracking results with weighted P-N learning. In Figure 2, the left and middle images are the ground truth and tracking results with weighted P-N learning, and the right images are tracking results based on P-N learning.

### 3.2. Constraints

In P-N learning, a constraint can be arbitrary function, especially two categories of constraints which we term P and N. P-constrains recognize samples which are labeled negative by the classifier, yet constraints need a positive label. P-constraints add *n* ^+^(*k*) samples to the training set in iteration *k*. Similarly, N-constraints are employed to identify samples classified as positive but constraints require negative label. In iteration *k*, N-constraints insert *n* ^−^(*k*) samples to training set.

In iteration *k*, the error of a classifier is represented by a number of false positives *α*(*k*) and a number of false negatives *β*(*k*). Let *n*_*c*_^+^(*k*) be the number of samples for which the label is correctly changed to positive in iteration *k* by P-constraints, and *n*_*f*_^+^(*k*) is then the number of samples for which the label is incorrectly changed to positive in iteration *k*. Hence, P-constraints change *n* ^+^(*k*) = *n*_*c*_^+^(*k*) + *n*_*f*_^+^(*k*) samples to positive. Similarly, N-constraints change *n* ^−^(*k*) = *n*_*c*_^−^(*k*) + *n*_*f*_^−^(*k*) samples to negative, where *n*_*c*_^−^(*k*) and *n*_*f*_^−^(*k*) are correct and false assignments. The errors of classifier can be represented as the following formulations:

(5) $$\begin{array}{c} \alpha \left(k+1\right)=\alpha \left(k\right)-n_{c}^{-}\left(k\right)+n_{f}^{+}\left(k\right), \end{array}$$

(6) $$\begin{array}{c} \beta \left(k+1\right)=\beta \left(k\right)-n_{c}^{+}\left(k\right)+n_{f}^{-}\left(k\right). \end{array}$$

Equation (5) demonstrates that false positives *α*(*k*) decrease if *n*_*c*_^−^(*k*) > *n*_*f*_^+^(*k*). In the similar way, false negatives *β*(*k*) decrease if *n*_*c*_^+^(*k*) > *n*_*f*_^−^(*k*). To analyze the convergence of learning process, a model needs to be developed that relates the performance of P-N constraints to *n*_*c*_^+^(*k*), *n*_*c*_^−^(*k*), *n*_*f*_^+^(*k*), and *n*_*f*_^−^(*k*).

The performance of P-N constraints is represented by four indexes, P-precision *P* ^+^, P-recall *R* ^+^, N-precision *P* ^−^, and N-recall *R* ^−^, determined by the following formulation:

(7) $$\begin{array}{c} P{\left(k\right)}^{+}=\frac{n_{c}^{+}\left(k\right)}{n_{c}^{+}\left(k\right)+n_{f}^{+}\left(k\right)},\\ R{\left(k\right)}^{+}=\frac{n_{c}^{+}\left(k\right)}{\beta \left(k\right)},\\ P{\left(k\right)}^{-}=\frac{n_{c}^{-}\left(k\right)}{n_{c}^{-}\left(k\right)+n_{f}^{-}\left(k\right)},\\ R{\left(k\right)}^{-}=\frac{n_{c}^{-}\left(k\right)}{\alpha \left(k\right)}. \end{array}$$

According to formulation (7), it is easy to get

(8) $$\begin{array}{c} n_{c}^{+}\left(k\right)=R{\left(k\right)}^{+}\cdot \beta \left(k\right),\\ n_{f}^{+}\left(k\right)=\frac{1-P{\left(k\right)}^{+}}{P{\left(k\right)}^{+}}\cdot R{\left(k\right)}^{+}\cdot \beta \left(k\right),\\ n_{c}^{-}\left(k\right)=R{\left(k\right)}^{-}\cdot \alpha \left(k\right),\\ n_{f}^{-}\left(k\right)=\frac{1-P{\left(k\right)}^{-}}{P{\left(k\right)}^{-}}\cdot R{\left(k\right)}^{-}\cdot \alpha \left(k\right). \end{array}$$

By combining formulations (5), (6), and (8), we can obtain new formulations:

(9) $$\begin{array}{c} \alpha \left(k+1\right)=\left(1-R{\left(k\right)}^{-}\right)\cdot \alpha \left(k\right)+\frac{1-P{\left(k\right)}^{+}}{P{\left(k\right)}^{+}}\cdot R{\left(k\right)}^{+}\cdot \beta \left(k\right),\\ \beta \left(k+1\right)=\frac{1-P{\left(k\right)}^{-}}{P{\left(k\right)}^{-}}\cdot R{\left(k\right)}^{-}\cdot \alpha \left(k\right)+\left(1-R{\left(k\right)}^{+}\right)\cdot \beta \left(k\right). \end{array}$$

After defining state vector $\vec{x}\left(k\right)={\left[\begin{array}{cc} \alpha \left(k\right) & \beta \left(k\right) \end{array}\right]}^{T}$ and transition matrix **M** as the following,

(10) $$\begin{array}{c} M=\left[\begin{array}{cc} 1-R{\left(k\right)}^{-} & \frac{1-P{\left(k\right)}^{+}}{P{\left(k\right)}^{+}}\cdot R{\left(k\right)}^{+}\\ \frac{1-P{\left(k\right)}^{-}}{P{\left(k\right)}^{-}}\cdot R{\left(k\right)}^{-} & 1-R{\left(k\right)}^{+} \end{array}\right], \end{array}$$

hence formulation (9) can be rewritten as the following formulation:

(11) $$\begin{array}{c} \vec{x}\left(k+1\right)=M\vec{x}\left(k\right). \end{array}$$

According to [27], formulation (11) is a recursive equation that is related to a discrete dynamical system. Based on the theory of dynamical systems, the state vector $\vec{x}$ converges to zero if eigenvalues *λ*_1_ and *λ*_2_ of the transition matrix **M** meet the condition *λ*_1_ < 1 and *λ*_2_ < 1. As pointed in [17], the performance of classifier will be improved constantly, only if the two eigenvalues of transition matrix **M** are smaller than one.

### 3.3. Object Detecting

In previous subsections, we illustrate the weighted P-N learning method. In this subsection, a classifier will be developed to detect the object. Scanning window strategy is utilized to detect the object in [17]. Similarly, we use this method to detect the object.

**Figure 3:**
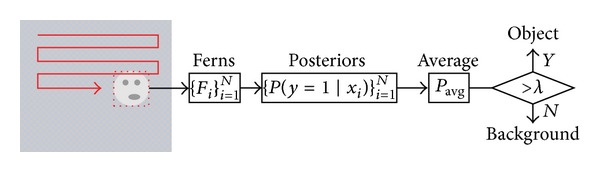
Object detection based on scanning window strategy and randomized forest classifier. The setting of the detector is as follows: 10,000 windows are scanned, 10 ferns per window.

In this paper, the randomized forest classifier [28] is adopted. For each input subwindow, classifier consists of *N* ferns. Each fern *i* computes the input patch resulting in feature vector *x*_*i*_, which is used to obtain posterior probability *P*(*y* = 1∣*x*_*i*_). The following formulation is defined to discriminate input patch:

(12) $$\begin{array}{c} P_{\text{avg}}>\lambda \;\text{object},\\ P_{\text{avg}}\leq \lambda \;\text{background}, \end{array}$$

where *P*_avg_ = ∑_*i*=1_^*N*^ *P*(*y* = 1∣*x*_*i*_) denotes the average of all posteriors and *λ* is the threshold which is set to 0.4 in all experiments. The detection process can be illustrated in Figure 3. Actually, *W*2_*j*_^+^ = *P*_avg_ and *W*2_*j*_^−^ = 1 − *P*_avg_. Feature vector is represented by 2-bit Binary Patterns [25] because of their invariance to illumination and efficient multiscale implementation using integral image. In fact, the posteriors *P*(*y* = 1∣*x*_*i*_) represent the parameter *θ* of the classifier and are estimated incrementally through the entire learning process. Each leafnode of fern records the number of positive *p* and negative *n* samples changed into it during iteration. The posteriors are then estimated by the following formulation:

(13) $$\begin{array}{c} P\left(y=1\;|\;x_{i}\right)=\frac{p}{p+n}\;\text{leaf is not empty,}\\ P\left(y=1\;|\;x_{i}\right)=0\;\text{leaf is empty.} \end{array}$$

The classifier is initialized in first frame, and posteriors are initialized to zero and renewed by 500 positive samples produced by affine warping of the selected patch [1]. The classifier is then evaluated on all the patches. In this paper, detections far from the selected patch represent the negative samples and update the posteriors.

## 4. Tracking

**Figure 4:**
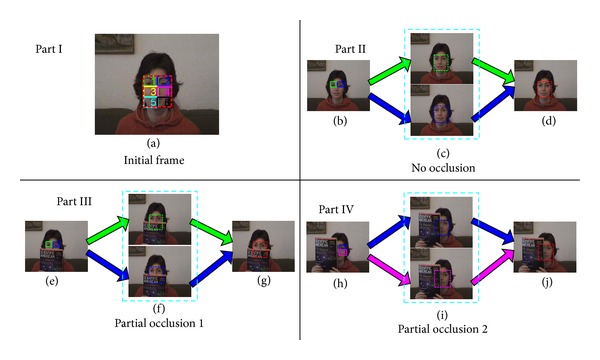
Illustration of tracking process. Part (I): segmentation of object in initial frame (first frame). Object is divided into six blocks. Part (II): tracking object without occlusion. Green block and blue block are selected as LFBs in image (b). Green and blue dotted line rectangles in image (c) are the tracking results of LFBs, and the red dotted line rectangle in image (d) represents the location of object estimated by the tracking results of LFBs. Part (III): tracking object with occlusion yet valid LFBs. Essentially, this process is similar to part (II). Occlusion does not affect the tracking. Part (IV): tracking object with occlusion with invalid LFB. In this part, green LFB is occluded and therefore invalid. We replace it with another new valid LFB, namely, the pink LFB, as shown in image (h). The object is then located in image (j) by tracking results of blue LFB and pink LFB in image (i).

In this paper, the object is represented by independent local feature blocks. The tracking task is then transformed into tracking each local feature block. We train classifier for each LFB with online weighted P-N learning, respectively, and then track each LFB independently within the framework of LK optical flow [21]. During tracking procedure, a real-time valid detection algorithm is utilized for each LFB. If certain LFB is invalid, it will be replaced with an unused block, which makes our tracker robust.  Figure 4 illustrates the principle of tracking.

### 4.1. Set of Local Feature Blocks

**Figure 5:**
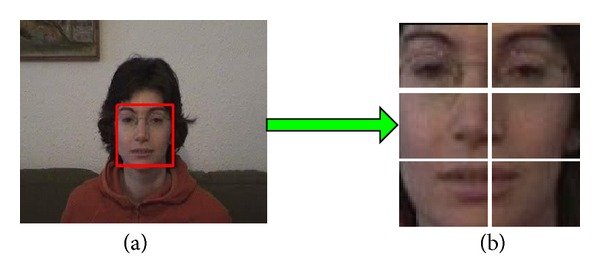
Uniform segmentation of object. Image (a) is object and image (b) is fragments.

Object is represented by LFBs, and thereby, object needs to be segmented into fragments. For simplicity, uniform segmentation is adopted in this paper as shown in Figure 5.

After segmentation, we select part blocks as LFBs. Assume object is divided into *K* blocks; then we can obtain a candidate set of LFB set *CB* = {*b*_1_, *b*_2_,…, *b*_*K*_} with *K* candidate local feature blocks. For candidate LFB *b*_*i*_, we compute its 2-bit Binary Patterns feature vector *x*_*i*_. Then, scanning window method is used to compute the similar likelihood between feature vectors of input patch *f*_*i*_ and *b*_*i*_, and the similarity is represented as *L*_*ij*_. *ML*_*i*_ = max⁡(*L*_*ij*_) represents the highest similarity between *b*_*i*_ and all input patches. Finally, local feature block set *SB* = {*sb*_1_, *sb*_2_,…, *sb*_*M*_} consists of *M*  (*M* < *K*) candidate local feature blocks, with smaller *ML*_*i*_.

### 4.2. Representations

In frame *t*, *B* ^*t*^ = (*C* ^*t*^, *R* ^*t*^, *W* ^*t*^, *H* ^*t*^) represents the object, where *C* ^*t*^ and *R* ^*t*^ are coordinates of center position and *W* ^*t*^ and *H* ^*t*^ are the sizes of object. *sb*_*k*_^*t*^ = (*c*_*k*_^*t*^, *r*_*k*_^*t*^, *w*_*k*_^*t*^, *h*_*k*_^*t*^) is the *k*th LFB, where *c*_*k*_^*t*^ and *r*_*k*_^*t*^ are coordinates of center location and *w*_*k*_^*t*^ and *h*_*k*_^*t*^ are the sizes of local feature block. *z*_*k*_^*t*^ = (*oc*_*k*_^*t*^, *or*_*k*_^*t*^, *rw*_*k*_^*t*^, *rh*_*k*_^*t*^) represents the offset of the *k*th LFB relative to object, where *oc*_*k*_^*t*^ and *or*_*k*_^*t*^ are the offset of center coordinates and *rw*_*k*_^*t*^ and *rh*_*k*_^*t*^ are the rations of sizes between object and the *k*th LFB. *z*_*k*_^*t*^ can be determined by the following formulation:

(14) $$\begin{array}{c} oc_{k}^{t}=C^{t}-c_{k}^{t},\\ or_{k}^{t}=R^{t}-r_{k}^{t},\\ rw_{k}^{t}=\frac{W^{t}}{w_{k}^{t}},\\ rh_{k}^{t}=\frac{H^{t}}{h_{k}^{t}}. \end{array}$$

### 4.3. Object Tracking

The tracked target is determined in initial frame (first frame) and segmented into fragments according to Section 4.1. Then, we select local feature blocks and compute *B* ^1^, *sb*_*k*_^1^, and *z*_*k*_^1^.

Assume current frame is *t*. Each LFB is corresponded with a classifier via weighted P-N learning, and then we track each LFB. For the *k*th LFB, *sb*_*k*_^*t*+1^ = (*c*_*k*_^*t*+1^, *r*_*k*_^*t*+1^, *w*_*k*_^*t*+1^, *h*_*k*_^*t*+1^) is used to represent its tracking result in frame *t* + 1. By combining tracking results of each LFB and its offset *z*_*k*_^*t*^, we can obtain the corresponding object *B*_*k*_^*t*+1^ = (*C*_*k*_^*t*+1^, *R*_*k*_^*t*+1^, *W*_*k*_^*t*+1^, *H*_*k*_^*t*+1^) via formulation the following formulation:

(15) $$\begin{array}{c} C_{k}^{t+1}=oc_{k}^{t}+c_{k}^{t+1},\\ R_{k}^{t+1}=or_{k}^{t}+r_{k}^{t+1},\\ W_{k}^{t+1}=rw_{k}^{t}\cdot w_{k}^{t+1},\\ H_{k}^{t+1}=rh_{k}^{t}\cdot h_{k}^{t+1}. \end{array}$$

**Figure 6:**
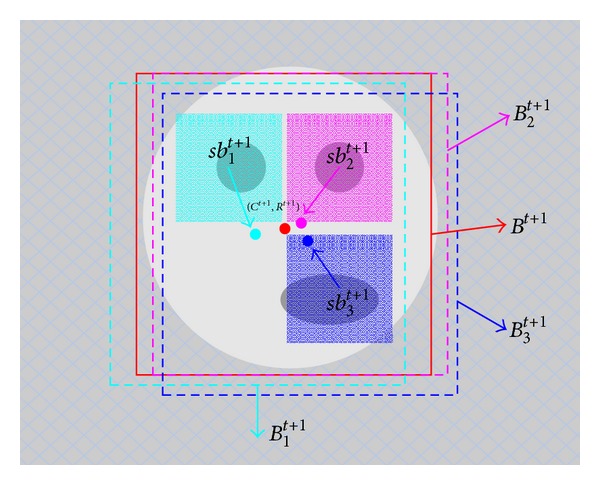
The process of locating object. The green, pink, and blue patches are the local feature blocks *sb*_1_^*t*+1^, *sb*_2_^*t*+1^, and *sb*_3_^*t*+1^, and the green, blue, and pink dotted line rectangles are the corresponding tracking results *B*_1_^*t*+1^, *B*_2_^*t*+1^, and *B*_3_^*t*+1^. The red solid line rectangle represents the final tracking result *B* ^*t*+1^, which is located by tracking results of local feature blocks.

Each LFB determines a related candidate object. Object finally can be located by the following formulation:

(16) $$\begin{array}{c} B^{t+1}=\frac{1}{M}\cdot \overset{M}{\underset{i=1}{\sum }}B_{i}^{t+1}, \end{array}$$

where *M* is the number of LFBs. The entire process can be explained by Figure 6. An adjustment is needed for offset of each LFB relative to object. We divide new object region *B* ^*t*+1^ into fragments and compute the new offset of each LFB with prior LFBs based on formulation (14). After this, classifier of each LFB needs to be retrained via weighted P-N learning.

Representation by LFBs has significant advantages. Firstly, compared with entire object, local feature block is more prone to recognition in background, and this guarantees the accuracy and stability of tracking. Besides, object is located by averaging all the tracking results of local feature blocks, which decreases tracking errors by counteracting positive and negative errors; therefore, the robustness of proposed algorithm is improved.

### 4.4. Updating Set of Local Feature Blocks

During the tracking process, update is essentially adaptive to complex environment variation. In this paper, the learning procedure is online, and then the main problem is how to handle the situation of local feature blocks being invalid. A strategy of replacing is adopted to solve this problem. During the tracking procedure, we make a real-time valid detection (in Section 3.3) for all local feature blocks. If *P*_avg_ ≤ *λ*, local feature block is invalid. When certain local feature block is invalid, it will be replaced with an appropriate block, which is selected from the outside of the LFB set.

Let *UB* be the unused block set, and *UB* = {*ub*_1_, *ub*_2_,…, *ub*_*P*_}, *P* = *K* − *M*, *UB* ⋃ *SB* = *CB*. In current frame *t*, LFB *sb*_*k*_^*t*^ from *SB* is invalid and needs to be replaced. We first segment object into blocks and obtain *UB*. For block *j* from *UB*, we compute similar likelihood *l*_*j*_^*t*^ = sim(*x*_*j*_^*t*^, *x*_*j*_^*q*^), where sim is function of computing similar likelihood, *x*_*j*_^*t*^ is feature vector of block *j* in frame *t*, and *x*_*j*_^*q*^ is feature vector of block *j* before it is used the last time in frame *q* (if block *j* is never used, *q* equals one). *ub*_*τ*_ is used to replace *sb*_*k*_^*t*^ via the following formulation:

(17) $$\begin{array}{c} ub_{\tau }=\arg\;\max l_{j}^{t}\left(ub_{j}\right). \end{array}$$

**Figure 7:**
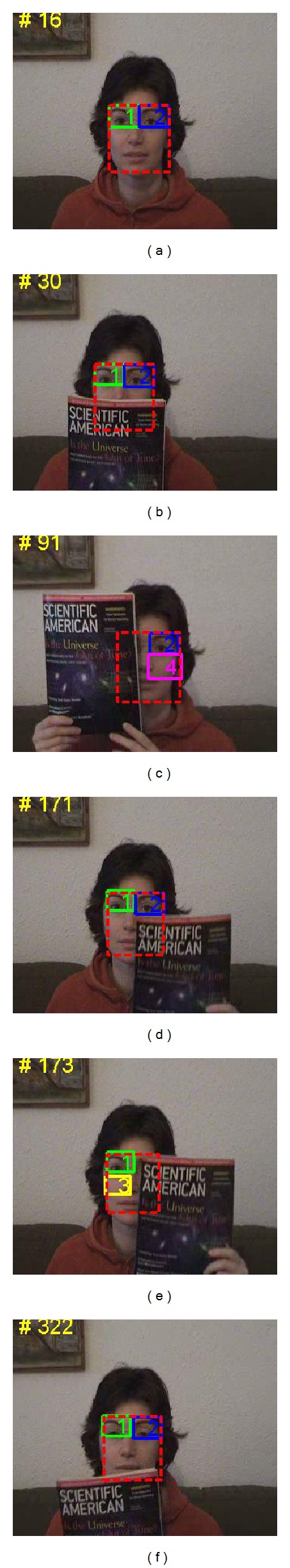
The process of updating. The object is located by LFBs in (a) without occlusion. When occlusion occurs, yet each LFB is valid, the object can be tracked by LFBs exactly, as shown in (b). If occlusion happens and certain LFB is invalid, then it will be replaced and target still can be successfully located, as shown in (c), (d), (e), and (f).

The whole update process can be illustrated as shown in Figure 7.

So far, we have introduced the overall procedure of the proposed tracking algorithm as shown in Algorithm 1.

## 5. Experimental Results

In order to evaluate the performance of our tracking algorithm, we test our tracker on thirteen challenging image sequences. These sequences cover most challenging situations in visual tracking as shown in Table 1. For comparison, we run six state-of-the-art tracking algorithms with the same initial position of object. These algorithms are *l*_1_ tracking [2], FG tracking [8], IVT tracking [3], MIL tracking [5], TLD tracking [26], and CT tracking [6] approaches. Some representative results are shown in this section.

### 5.1. Quantitative Comparison

We evaluate the above-mentioned trackers via overlapping rate [29] as well as center location error, and the comparing results are shown in Tables 2 and 3.

**Figure 8:**
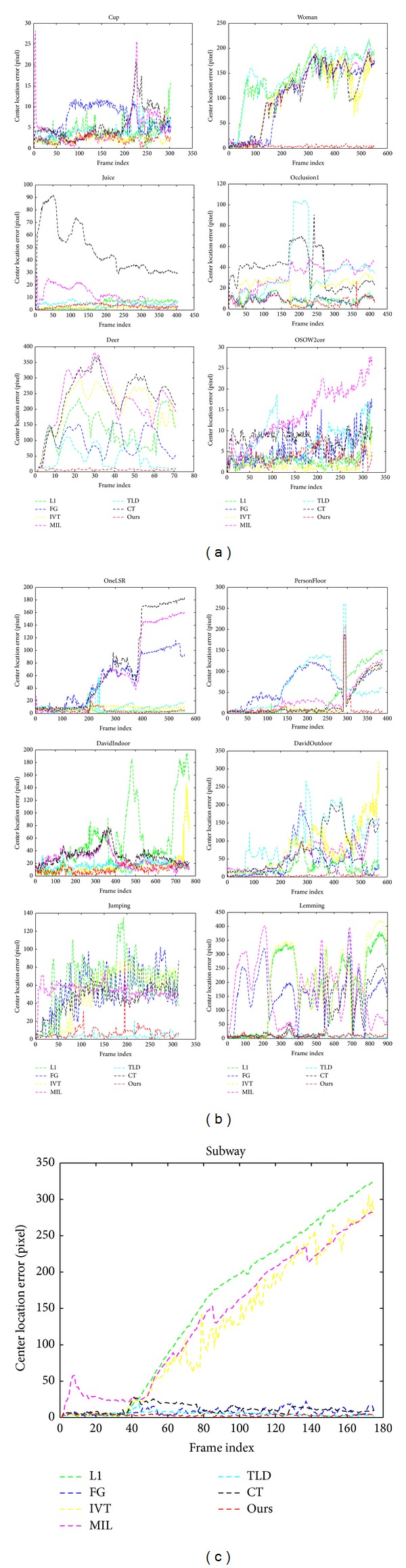
Quantitative evaluation of the trackers in terms of position errors (in pixels).

Figure 8 shows the center location error of utilized tracker on thirteen test sequences. Overall, the tracker proposed in this paper outperforms the state-of-the-art algorithms.

### 5.2. Qualitative Comparison

**Figure 9:**
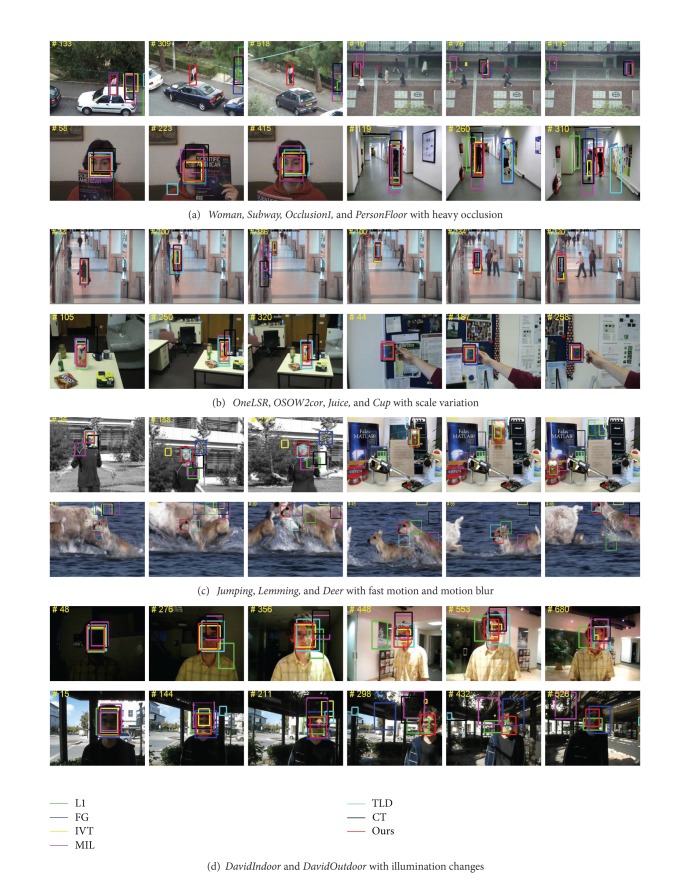
Tracking results on various challenging sequences.

*Heavy Occlusion*. Occlusion is one of the most common yet crucial issues in visual tracking. We test four image sequences (Woman, Subway, PersonFloor, and Occlusion1) characterized in severe occlusion or long-time partial occlusion.  Figure 9(a) demonstrates the robustness performance of proposed tracking method in handling occlusion. Object is represented by local feature blocks in proposed algorithm. When occlusion happens, other tracking algorithms cannot track object well because they are prone to update background into object. However, our tracker can employ a new unused block to replace the invalid local feature block when occlusion occurs, which can make local feature block set effective to continue tracking.

*Scale Variation*.  Figure 9(b) presents the tracking results on four image sequences (OneLSR, OSOW2cor, Juice, and Cup) with large scale variation, even more with slight rotation. Our tracker can tail object throughout the whole sequences, which can be attributed to the discriminative classifier based on weighted P-N learning. We also observe that local feature blocks can better represent object, which makes the tracker focus on the stable part of the object.

*Fast Motion and Motion Blur*.  Figure 9(c) demonstrates experimental results on three challenging sequences (Deer, Lemming, and Jumping). Because the target undergoes fast and abrupt motion, it is more prone to cause blur, which causes drifting problem. It is worth noticing that the suggested approach in this paper performs better than other algorithms. When motion blur occurs, our tracker can guarantee that the object's local feautres are still available. The advantages of using local feature blocks to represent object are shown incisively and vividly. By combining improved P-N learning and local feature blocks, we can obtain a discriminative classifier of stable object parts, which can locate the object. Then we track each local feature block, respectively, and determine the object based on tracking results of local feature blocks.

*Illumination Variation*. Illumination is a critical factor in visual tracking. Two typical image sequences (DavidIndoor and DavidOutdoor) are employed to test our tracker as shown in Figure 9(d). When illumination varies, some local regions of target are insensitive actually. Our tracker captures these insensitive regions to track local areas of object and further locate entire object via local tracking information.

## 6. Conclusions

In this paper, we propose a part-based visual tracking algorithm with online weighted P-N learning. An online P-N learning is presented by assigning weight (property weight and classification weight) to each sample in training sample set, which can decrease classification errors and can improve the discriminative power of classifier. Firstly, the target is segmented into fragments, and parts of them are chosen to be local feature blocks to represent object. We then train classifier for each LFB with weighted P-N learning, obtain the corresponding classifier, respectively, and track each LFB independently within the framework of LK optical flow. In addition, a substitute strategy is adopted to update dynamically the set of LFBs, which ensures robust tracking. Experimental results demonstrate that our algorithm outperforms state-of-the-art trackers. However, our algorithm fails to track object exactly in some scenes. If the tracked target is nonrigid and has an extremely heavy deformation or is fully occluded for long time, the performance of proposed tracker drops.

## Figures and Tables

**Algorithm 1:**
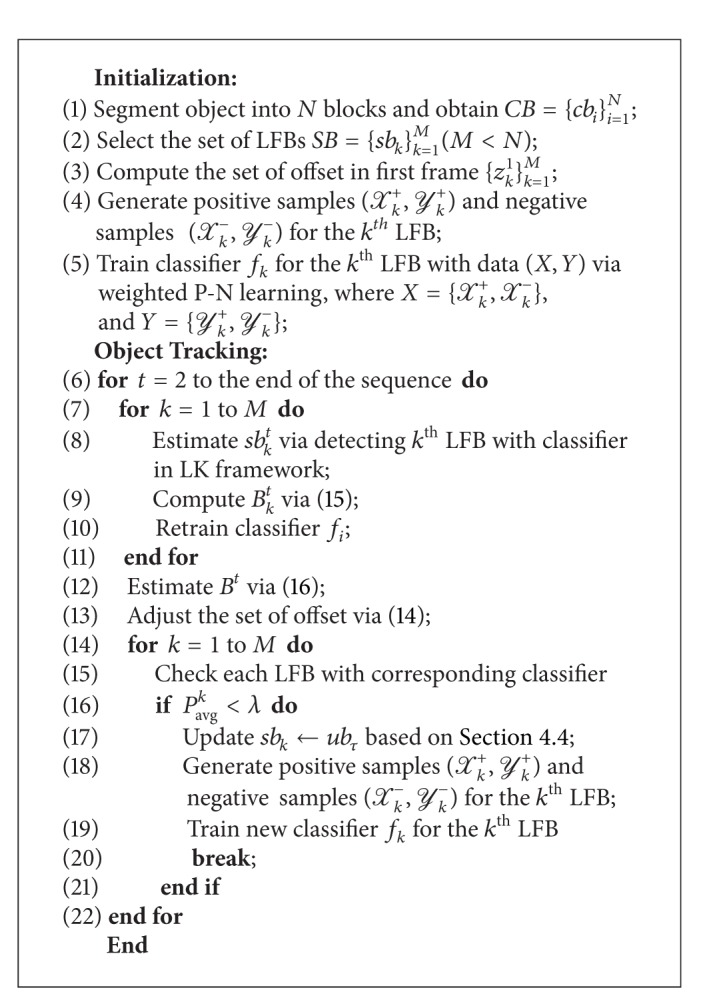
Tracking based on proposed method.

**Table 1:** The tracking sequences used in our experiments.

Sequence	Frames	Main challenges
Cup	303	Direction variation and scale variation
DavidIndoor	770	Illumination, scale variation, and pose change
DavidOutdoor	569	Illumination and scale variation
Deer	71	Fast motion, motion blur, and background clutter
Juice	404	Direction variation and scale variation
Jumping	313	Fast motion and motion blur
Lemming	900	Fast motion, motion blur, and occlusion
Occlusion1	415	Occlusion
OneLSR	559	Scale variation and occlusion
OSOW2cor	320	Scale variation and pose change
PersonFloor	387	Scale variation and occlusion
Subway	175	Occlusion and background clutter
Woman	551	Occlusion, scale variation, and pose change

**Table 2:** Center location errors (in pixels). The best result is shown in bold and the second best in italic fonts.

	*l*_1_	FG	IVT	MIL	TLD	CT	Ours
Woman	136.69	*106.21 *	110.46	114.02	148.66	107.74	** 3.77 **
DavidIndoor	57.52	—	*15.78 *	26.43	16.19	31.83	** 10.34 **
Cup	4.09	7.02	**2.23 **	4.78	3.86	5.62	* 2.47 *
Juice	4.39	—	**1.80 **	11.07	4.57	47.97	* 3.07 *
Deer	132.59	97.28	206.68	231.29	*45.83 *	240.36	**7.02 **
Lemming	181.13	164.87	192.12	168.87	**8.19 **	73.28	*10.14 *
OneLSR	*4.87 *	54.70	8.43	65.48	10.50	76.47	** 3.95 **
PersonFloor	37.63	68.15	27.06	37.13	62.94	*26.89 *	**9.52 **
Subway	153.60	8.06	124.60	137.86	*5.26 *	11.50	** 3.19 **
OSOW2cor	*3.06 *	5.22	**1.62 **	13.83	7.50	8.08	3.97
DavidOutdoor	*26.50 *	45.87	75.86	59.77	102.20	73.72	** 3.71 **
Occlusion1	8.85	*8.60 *	21.81	31.91	35.43	37.96	** 7.11 **
Jumping	64.23	52.34	50.18	54.03	**4.06 **	45.11	*6.87 *

Average	62.70	56.21	64.51	73.57	*35.01 *	60.50	**5.78 **

**Table 3:** Overlapping rate. Bold fonts indicate the best performance while the italic fonts indicate the second best ones.

	*l*_1_	FG	IVT	MIL	TLD	CT	Ours
Woman	0.06	*0.19 *	0.14	0.15	0.04	0.15	** 0.72 **
DavidIndoor	0.21	—	0.3	0.45	**0.52 **	0.39	*0.47 *
Cup	0.64	0.67	0.61	**0.77 **	0.63	*0.73 *	0.62
Juice	*0.73 *	—	**0.79 **	0.64	0.66	0.07	0.69
Deer	0.04	0.07	0.03	0.04	*0.34 *	0.04	** 0.65 **
Lemming	0.19	0.05	0.19	0.07	*0.64 *	0.43	** 0.7 **
OneLSR	*0.54 *	0.24	0.3	0.26	0.45	0.26	** 0.56 **
PersonFloor	0.47	0.25	0.53	0.47	0.21	*0.6 *	**0.64 **
Subway	0.15	0.61	0.17	0.09	**0.68 **	0.5	* 0.67 *
OSOW2cor	0.59	0.43	**0.63 **	0.39	0.5	0.4	* 0.62 *
DavidOutdoor	0.35	*0.42 *	0.17	0.3	0.08	0.31	**0.62 **
Occlusion1	**0.71 **	**0.72 **	0.5	0.55	0.48	0.41	0.66
Jumping	0.07	0.1	0.17	0.01	**0.78 **	0.07	*0.75 *

Average	0.37	0.36	0.35	0.32	*0.46 *	0.33	**0.64 **

## References

[1] Wu Y, Lim J, Yang MH Online object tracking: a benchmark.

[2] Mei X, Ling H Robust visual tracking using *ℓ* 1 minimization.

[3] Ross DA, Lim J, Lin RS, Yang MH (2008). Incremental learning for robust visual tracking. *International Journal of Computer Vision*.

[4] Comaniciu D, Ramesh V, Meer P (2003). Kernel-based object tracking. *IEEE Transactions on Pattern Analysis and Machine Intelligence*.

[5] Babenko B, Belongie S, Yang M Visual tracking with online multiple instance learning.

[6] Zhang K, Zhang L, Yang MH Real-time compressive tracking.

[7] Jia X, Wang D, Lu H Fragment-based tracking using online multiple kernel learning.

[8] Adam A, Rivlin E, Shimshoni I Robust fragments-based tracking using the integral histogram.

[9] Shahed Nejhum SM, Ho J, Yang MH Visual tracking with histograms and articulating blocks.

[10] Wang S, Lu H, Yang F, Yang M Superpixel tracking.

[11] Yao R, Shi Q, Shen C, Zhang Y, van den Hengel A Part-based visual tracking with online latent structural learning.

[12] Yu Q, Dinh BT, Medioni G Online tracking and reacquisition using co-trained generative and discriminative trackers.

[13] Zhang T, Ghanem B, Liu S, Ahuja N Robust visual tracking via multi-task sparse learning.

[14] Kwon J, Lee KM Visual tracking decomposition.

[15] Bao C, Wu Y, Ling H, Ji H Real time robust L1 tracker using accelerated proximal gradient approach.

[16] Liu B, Huang J, Yang L, Kulikowsk C Robust tracking using local sparse appearance model and K-selection.

[17] Kalal Z, Matas J, Mikolajczyk K P-N learning: bootstrapping binary classifiers by structural constraints.

[18] Grabner H, Leistner C, Bischof H Semisupervised on-line boosting for robust tracking.

[19] Grabner H, Matas J, Van Gool L, Cattin P Tracking the invisible: Learning where the object might be.

[20] Zhang K, Song H (2013). Real-time visual tracking via online weighted multiple instance learning. *Pattern Recognition*.

[21] Lucas BD, Kanade T An iterative image registration technique with an application to stereo vision.

[22] Wang F, Yu S, Yang J A novel fragments-based tracking algorithm using mean shift.

[23] Tang F, Brennan S, Zhao Q, Tao H Co-tracking using semi-supervised support vector machines.

[24] Kalal Z, Mikolajczyk K, Matas J Face-TLD: tracking-learning-detection applied to faces.

[25] Kalal Z, Matas J, Mikolajczyk K Online learning of robust object detectors during unstable tracking.

[26] Kalal Z, Mikolajczyk K, Matas J (2012). Tracking-learning-detection. *IEEE Transactions on Pattern Analysis and Machine Intelligence*.

[27] Zhou K, Doyle JC, Glover K (1996). *Robust and Optimal Control*.

[28] Breiman L (2001). Random forests. *Machine Learning*.

[29] Everingham M, Gool LV, Williams C Partbased visual trackin g with online latent structural learning.

